# Selective inhibition of carbonic anhydrase IX and XII by coumarin and psoralen derivatives

**DOI:** 10.1080/14756366.2021.1887171

**Published:** 2021-02-18

**Authors:** Rita Meleddu, Serenella Deplano, Elias Maccioni, Francesco Ortuso, Filippo Cottiglia, Daniela Secci, Alessia Onali, Erica Sanna, Andrea Angeli, Rossella Angius, Stefano Alcaro, Claudiu T. Supuran, Simona Distinto

**Affiliations:** a Department of Life and Environmental Sciences, University of Cagliari, Monserrato, Italy; b Dipartimento di Scienze della Salute, Università Magna Graecia di Catanzaro, Catanzaro, Italy; c Dipartimento NEUROFARBA, Sezione di Scienze Farmaceutiche, Università degli Studi di Firenze, Sesto Fiorentino, Italy; d Laboratorio NMR e Tecnologie Bioanalitiche, Sardegna Ricerche, Pula, Italy

**Keywords:** hCAi, tumour, coumarin, psoralen, docking

## Abstract

A small library of coumarin and their psoralen analogues **EMAC10157a-b-d-g** and **EMAC10160a-b-d-g** has been designed and synthesised to investigate the effect of structural modifications on their inhibition ability and selectivity profile towards carbonic anhydrase isoforms I, II, IX, and XII. None of the new compounds exhibited activity towards hCA I and II isozymes. Conversely, both coumarin and psoralen derivatives were active against tumour associated isoforms IX and XII in the low micromolar or nanomolar range of concentration. These data further corroborate our previous findings on analogous derivatives, confirming that both coumarins and psoralens are interesting scaffolds for the design of isozyme selective hCA inhibitors.

## Introduction

The development of cancer is a complex multifactorial process, involving many cellular adaptations and signal transduction pathways^1–4^. In solid tumours, cancer cells must survive in a low oxygen concentration environment, due to the rapid cellular proliferation and to the impossibility to promptly supply an adequate vascularisation^5^^,^^6^. Indeed, many pathways are involved in the hypoxia survival mechanism^7–11^ and they all concur in helping cancer cells to escape from apoptosis. These pathways have been investigated in depth and might be inhibited by relatively new classes of anticancer drugs, to contrast the angiogenesis process, such as VEGFR (sunitinib, sorafenib), VEGF directed monoclonal antibodies (bevacizumab), and mTOR (everolimus, temsirolimus) inhibitors^12–17^. In this contest, the key role of human carbonic anhydrases, a class of metalloproteins that catalyse the reversible conversion of carbon dioxide to bicarbonate and protons^18–20^, has been outlined. In particular, two membrane isoforms, namely hCA IX and XII, are mainly involved in cancer proliferation and invasion^21–31^. Not surprisingly, several inhibitors of membrane bounded hCA isozymes, with diverse structures and mechanisms of action, have been designed and investigated so far. In this respect, both synthetic and natural coumarin derivatives have already demonstrated to possess high selectivity and activity towards specific hCA isozymes^32–36^. Moreover, their interaction and binding mode on this class of metalloenzymes have been investigated in depth^37^^,^^38^. Besides, it should be considered that coumarins have been reported to interact with several cancer druggable targets. In particular, coumarin derivatives have shown a variety of biological activities such as CK2 inhibitors^39^, EGFR^40^, PI3K-AKT-mTOR signalling inhibitors^41–43^. Furthermore, their anticancer potential, tumour targets, diverse mechanisms of action as well as their advantages and disadvantages have been recently reviewed^44^. In continuation with our previous work and prompted by these considerations, to further explore the influences of structural modifications on the coumarin and psoralene core on the activity and selectivity towards membrane-bound hCA isozyme, we have designed and synthesised a small library of methyl-2-[4-methyl-2-oxo-7–(2-oxo-2-arylethoxy)-8-propylchromen-3-yl]acetate and 2–(5-methyl-7-oxo-3-aryl-9-propyl-7H-furo[3,2-g]chromen-6-yl)acetic acid derivatives were a propyl group in the position 8 or 9 has been introduced compared to the previously synthesised derivatives.

## Methods

### Materials and apparatus

Starting materials and reagents were obtained from commercial suppliers and were used without purification. All melting points were determined on a Stuart SMP11 melting points apparatus and are uncorrected. Melting points, the yield of reactions, and analytical data of derivatives **EMAC10157a-b-d-g** and **EMAC10160a-b-d-g** are reported in Table 1.

**Table 1.** Chemical, analytical, and physical data of derivatives **EMAC10157 a-b-d-g** and **EMAC10160 a-b-d-g**.

Compound	R	C, %; H, %		M.P., °C	Yield, %^a^	Aspect
		Calc.	Found			
**EMAC10157a**	4-CH_3_	C, 71.07; H, 6.20	C, 71.10; H, 6.19	132–133	80	White crystals
**EMAC10157b**	4-OCH_3_	C, 68.48; H, 5.98	C, 68.74; H, 5.97	127–130	89	Pale brown crystals
**EMAC10157d**	4-F	C, 67.60; H, 5.44	C, 67.79; H, 5.45	164–166	90	White crystals
**EMAC10157g**	4-C_6_H_5_	C, 74.36; H, 5.82	C, 74.55; H, 5.45	138–140	71	White crystals
**EMAC10160a**	4-CH_3_	C, 74.24; H, 5.98	C, 74.19; H, 5.99	226–229	98	Grey crystals
**EMAC10160b**	4-OCH_3_	C, 71.41; H, 5.75	C, 71.68, H, 5.76	202–205	97	Green crystals
**EMAC10160d**	4-F	C, 70.58; H, 5.18	C, 70.30; H, 5.16	135–139	92	Grey crystal
**EMAC10160g**	4-C_6_H_5_	C, 77.24; H, 5.62	C, 77.52; H, 5.60	248–250	97	Pale brown crystal

a Yields are referred to the last step of the synthetic pathway.

^1^H-NMR and ^13^C-NMR spectra (Table 2) were registered on a Bruker AMX 400 MHz (chemical shifts in *δ* values) operating at 400 MHz and 100 MHz, respectively. All samples were measured in DMSO. Chemical shifts are reported referenced to the solvent in which they were measured. Coupling constants *J* are expressed in hertz (Hz). Elemental analyses were obtained on a Perkin–Elmer 240 B microanalyser. Analytical data of the synthesised compounds are in agreement within ±0.4% of the theoretical values. TLC chromatography was performed using silica gel plates (Merck F 254), spots were visualised by UV light.

**Table 2.** ^1^H NMR and ^13^C NMR data of derivatives **EMAC10157a-b-d-g** and **EMAC10160a-b-d-g**.

Compound	^1^H NMR and ^13^C NMR *δ* (ppm)
**EMAC10157a**	1H NMR (400 MHz, DMSO) *δ* 7.93 (d, 2H, *J* = 8), 7.61 (d, 1H, *J* = 9.2), 7.39 (d, 2H, *J* = 8), 7.00 (d, 1H, *J* = 8.8), 5.74 (s, 2H), 3.68 (s, 2H), 3.62 (s, 3H), 2.83–2.79 (m, 2H), 2.41 (s, 3H), 2.37 (s, 3H), 1.64–1.55 (m, 2H), 0.95–0.91 (t, 3H). 13C NMR (100 MHz, DMSO) *δ* 193.70, 170.64, 160.88, 158.24, 150.61, 149.69, 144.37, 131.78, 129.34 (2C), 127.96 (2C), 123.92, 116.97, 115.87, 113.65, 108.77, 70.55, 51.82, 32.37, 24.34, 21.70, 21.22, 15.10, 13.96.
**EMAC10157b**	1H NMR (400 MHz, DMSO) *δ* 8.01 (d, 2H, *J* = 8.8), 7.61 (d, 1H, *J* = 8), 7.09 (d, 2H, *J* = 8), 6.98 (d, 1H, *J* = 8), 5.70 (s, 2H), 3.86 (s, 3H), 3.68 (s, 2H), 3.62, (s, 3H), 2.82–2.79 (m, 2H), 2.36 (s, 3H), 1.64–1.55 (m, 2H), 0.95–0.91 (t, 3H) 13C NMR (100 MHz, DMSO) *δ* 192.48, 170.64, 163.61, 160.89, 158.29, 150.60, 149.69, 130.23 (2C), 127.15 123.90, 116.96, 115.84, 114.05 (2C), 113.61, 108.76, 70.35, 55.60, 51.81, 32.36, 24.34, 21.70, 15.09, 13.96.
**EMAC10157d**	1H NMR (400 MHz, DMSO) *δ* 8.13–8.10 (m, 2H), 7.62 (d, 1H, *J* = 9.2), 7.45–7.40 (m, 2H), 7.03 (d, 1H, *J* = 9.2), 5.77 (s, 2H), 3.68 (s, 2H), 3.62 (s, 3H), 2.83–2.79 (m, 2H), 2.37 (s, 3H), 1.64–1.55 (m, 2H), 0.95–0.91 (t, 3H). 13C NMR (100 MHz, DMSO) *δ* 192.90, 170.65, 166.59, 164.08, 160.89, 158.15, 150.60, 149.70, 131.01 (2C), 123.94, 116.98, 116.01, 115.91 (2C), 113.70, 108.79, 70.55, 51.82, 32.36, 24.33, 21.70, 15.10, 13.95.
**EMAC10157g**	1H NMR (400 MHz, DMSO) *δ* 8.12 (d, 2H, *J* = 8.4), 7.88 (d, 2H, *J* = 8.4), 7.78 (d, 2H, *J* = 7.2), 7.65–7.61 (m, 1H), 7.53 (t, 2H, *J* = 7.2), 7.45 (t, 1H, *J* = 7.2), 7.06–7.02 (m, 1H), 5.82 (s, 2H), 3.68 (s, 2H), 3.62 (s, 3H), 2.85–2.81 (m, 2H),2.37 (s, 3H), 1.64–1.58 (m, 2H), 0.96–0.92 (t, 3H). 13C NMR (100 MHz, DMSO) *δ* 193.78, 170.63, 160.88, 158.22, 150.62, 149.68, 145.17, 138.77, 133.07, 129.10 (2C), 128.61 (2C), 127.02 (2C), 126.96 (2C), 123.94, 116.99, 115.90, 113.68, 108.81, 73.94, 70.69, 51.81, 32.37, 24.37, 21.73, 15.11, 13.98.
**EMAC10160a**	1H NMR (400 MHz, DMSO) *δ* 12.47 (bs, 1H), 8.41 (s, 1H), 8.03 (s, 1H), 7.69 (d, 2H, *J* = 8), 7.34 (d, 2H, *J* = 7.6),3.65 (s, 2H), 3.05–3.01 (m, 2H), 2.51 (s, 3H), 2.38 (s, 3H), 1.78–1.69 (m, 2H), 0.97–0.94 (t, 3H). 13C NMR (100 MHz, DMSO) *δ* 171.49, 160.72, 154.93, 149.65, 147.36, 143.62, 137.11, 129.72 (2C), 127.82, 127.11 (2C), 122.21, 121.45, 117.83, 116.71, 114.20, 112.93, 32.90, 24.85, 21.93, 20.79, 15.64, 13.83.
**EMAC10160b**	1H NMR (400 MHz, DMSO) *δ* 12.47 (bs, 1H), 8.37 (s, 1H), 8.01 (s,1H), 7.73 (d, 2H, *J* = 8), 7.10 (d, 2H, *J* = 8.8), 3.82 (s, 3H), 3.65 (s, 2H), 3.04–3.01 (m, 2H), 2.51 (s, 3H), 1.71–1.68 (m, 2H), 0.97–0.94 (t, 3H). 13C NMR (100 MHz, DMSO) *δ* 171.50, 160.73, 158.92, 154.89, 149.66, 147.34, 143.17, 128.47 (2C), 123.00, 122.31, 121.18, 117.79, 116.66, 114.62 (2C), 114.16, 112.89, 55.17, 32.90, 24.84, 21.92, 15.65, 13.82.
**EMAC10160d**	1H NMR (400 MHz, DMSO) *δ* 12.47 (bs, 1H), 8.46 (s, 1H), 8.03 (s, 1H), 7.87–7.85 (m, 2H), 7.39–7.35 (m, 2H), 3.65 (s, 2H), 3.05–3.01 (m, 2H), 2.52 (s, 3H), 1.78–1.69 (m, 2H), 0.97–0.94 (t, 3H). 13C NMR (100 MHz, DMSO) *δ* 171.48, 162.91, 160.68, 160.51, 154.88, 149.67, 147.42, 129.23, 129.23, 127.21, 121.97, 120.59, 117.90, 116.81, 116.16, 115.94, 114.16, 112.98, 32.90, 27.45, 24.84, 21.92, 15.67, 13.82.
**EMAC10160g**	1H NMR (400 MHz, DMSO) *δ* 12.49 (bs, 1H), 8.54 (s, 1H), 8.13 (s, 1H), 7.92 (d, 2H, *J* = 8), 7.84 (d, 2H, *J* = 8.4), 7.75 (d, 2H, *J* = 7.2), 7.51 (t, 2H, *J* = 7.2), 7.40 (t, 1H, *J* = 7.2), 3.67 (s, 2H), 3.07–3.04 (m, 2H), 2.55 (s, 3H), 1.80–1.71 (m, 2H), 0.99–0.95 (t, 3H). 13C NMR (100 MHz, DMSO) *δ* 171.49, 160.71, 155.01, 149.69, 147.44, 144.21, 139.56, 139.42, 129.93, 129.01 (2C), 127.73 (2C), 127.59, 127.37 (2C), 126.54 (2C), 122.04, 121.13, 117.92, 116.84, 114.35, 113,03, 32.92, 24.88, 21.94, 15.71, 13.85.

### General procedure for the synthesis of compound EMAC10157 a-b-d-g and EMAC10160 a-b-d-g

#### Synthesis of methyl 2–(7-hydroxy-4-methyl-2-oxo-8-propyl-2H-chromen-3-yl)acetate

A mixture of propylresorcinol (1 eq.), dimethylacetylsuccinate (1 eq.) and sulphuric acid 98% (2.8 eq.) was vigorously stirred at room temperature. The progression of the reaction was monitored by TLC, using ethyl acetate/n-hexane 2:1. After 30 min a homogeneous sticky solid was obtained which was dissolved in methanol and poured into ice water. The mixture was stirred until ice melting and then filtered off to obtain a light yellow solid. The crude product was washed with ethyl ether giving a white powder that was crystallised from methanol.

#### Synthesis of methyl-2-[4-methyl-2-oxo-7–(2-oxo-2-arylethoxyi)-8-propylchromen-3-yl]acetate (EMAC 10157 a-b-d-g)

A hot solution of methyl 2–(7-hydroxy-4-methyl-2-oxo-8-propyl-2H-chromen-3-yl)acetate (1 eq.) in dry acetone was treated with K_2_CO_3_ (2.5 eq.), stirred vigorously, and treated with the appropriate α-haloketone (1 eq.). The reaction mixture was heated to reflux and stirred for 1–5 h (course of the reaction monitored by TLC using ethyl acetate/n-hexane 5:1). When the reaction was completed, it was cooled at 0 °C and the solution acidified with HCl conc. The resulting precipitate was filtered off and crystallised if necessary.

#### Synthesis of 2–(5-methyl-7-oxo-3-aryl-9-propyl-7H-furo[3,2-g]chromen-6-yl)acetic acid (EMAC10160 a-b-d-g)

A solution or suspension of coumarin (1 eq., **EMAC10157 a-b-d-g**) in propan-2-ol was treated with NaOH solution (4 eq., 1 N). The reaction mixture was heated for 3–4 h, obtaining a dark solution. The solution was cooled to room temperature and poured into ice water. Concentrated HCl was added to the solution, obtaining a suspension that was filtered and crystallised.

Analytical and spectral data of compounds **EMAC10157 a-b-d-g** and **EMAC10160 a-b-d-g** are reported in Tables 1 and 2. ^1^H-NMR and ^13^C-NMR spectra are reported in Supplementary material (Figures S1–S16).

### Biological activity

#### Carbonic anhydrase inhibition assay

The CA catalysed CO_2_ hydration/inhibition was measured by using a stopped-flow instrument (Applied Photophysics, Oxford, U.K.) as the method described earlier^45^. Initial rates of the CA-catalysed CO_2_ hydration reaction were followed for 10–100 s. The CO_2_ concentrations ranged from 1.7 to 17 mM for the determination of the inhibition constants. For each inhibitor, at least six traces of the initial 5–10% of the reaction were used for assessing the initial velocity. The uncatalyzed rates were subtracted from the total observed rates. Stock solutions of inhibitors (10 mM) and dilutions up to 0.01 nM were prepared in distilled-deionized water. Inhibitor and enzyme solutions were preincubated together for 15 min at room temperature prior to assay, in order to allow for the formation of the E–I complex. The inhibition constants were obtained by non-linear least-squares methods using PRISM 3 as reported earlier, and represent the mean from at least three different determinations^46–48^. hCA I, hCA II, hCA IX (catalytic domain), and hCA XII (catalytic domain) were recombinant proteins produced in-house using our standardised protocol and their concentration in the assay system was in the range of 3–10 nM (and even lower for highly effective, sub-nanomolar inhibitors)^46–48^.

### Molecular modelling

#### Ligand preparation

The ligands were built using the Maestro GUI^49^. The most stable conformation has been determined by molecular mechanics conformational analysis performed by Macromodel software version 9.2^50^. using the Merck Molecular Force Fields (MMFFs)^51^ and GB/SA water implicit solvation model^52^, Polak–Ribier Conjugate Gradient (PRCG) method, 5000 iterations, and a convergence criterion of 0.05 kcal/(mol Å). All the other parameters were left as default.

#### Protein preparation

The coordinates for hCA isoforms enzymes were taken from the RCSB Protein Data Bank^53^ (PDB codes 3k34^54^, for isoform II; 5fl4^55^, for isoform IX and 5msa, for isoform XII). The proteins were prepared by using the Maestro Protein Preparation Wizard^49^. Original water molecules were removed.

#### Docking protocol

Molecular docking studies were performed using QMPL workflow protocol^56^. Grids were defined around the refined structure by centring on crystallised ligands. The other settings were left as default.

#### Post docking protocol

To better consider, the induced fit phenomena, the most energy favoured generated complexes were fully optimised with the OPLS2005 force field in GB/SA implicit water. The optimisation process was performed setting 10,000 steps interactions up to the derivative convergence criterion equal to 0.05 kJ/(molÅ). The resulting complexes were considered for the binding modes graphical analysis with Pymol^57^ and Maestro.

## Results and discussion

**Figure 1.**
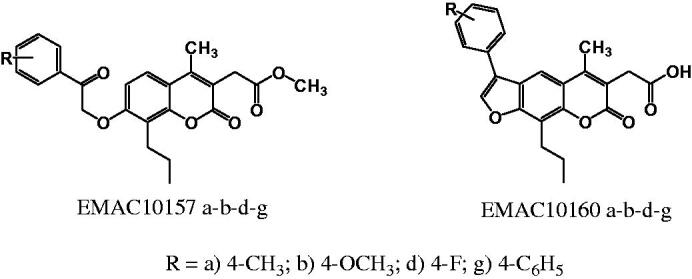
Newly synthesised coumarin and psoralen derivatives.

As a continuation of our ongoing research in the field of carbonic anhydrase and anticancer agents^58–62^ we have synthesised a new series of *methyl-2-[4-methyl-2-oxo-7–(2-oxo-2-arylethoxyi)-8-propylchromen-3-yl]acetate* and *2–(5-methyl-7-oxo-3-aryl-9-propyl-7H-furo[3,2-g]chromen-6-yl)acetic acid* to evaluate their activity and selectivity towards hCA isozymes and to gain information on their structure-activity relationships. The compounds are reported as **EMAC10157 a-b-d-g** and **EMAC10160 a-b-d-g** and their structures are illustrated in Figure 1.

**Figure 2.**
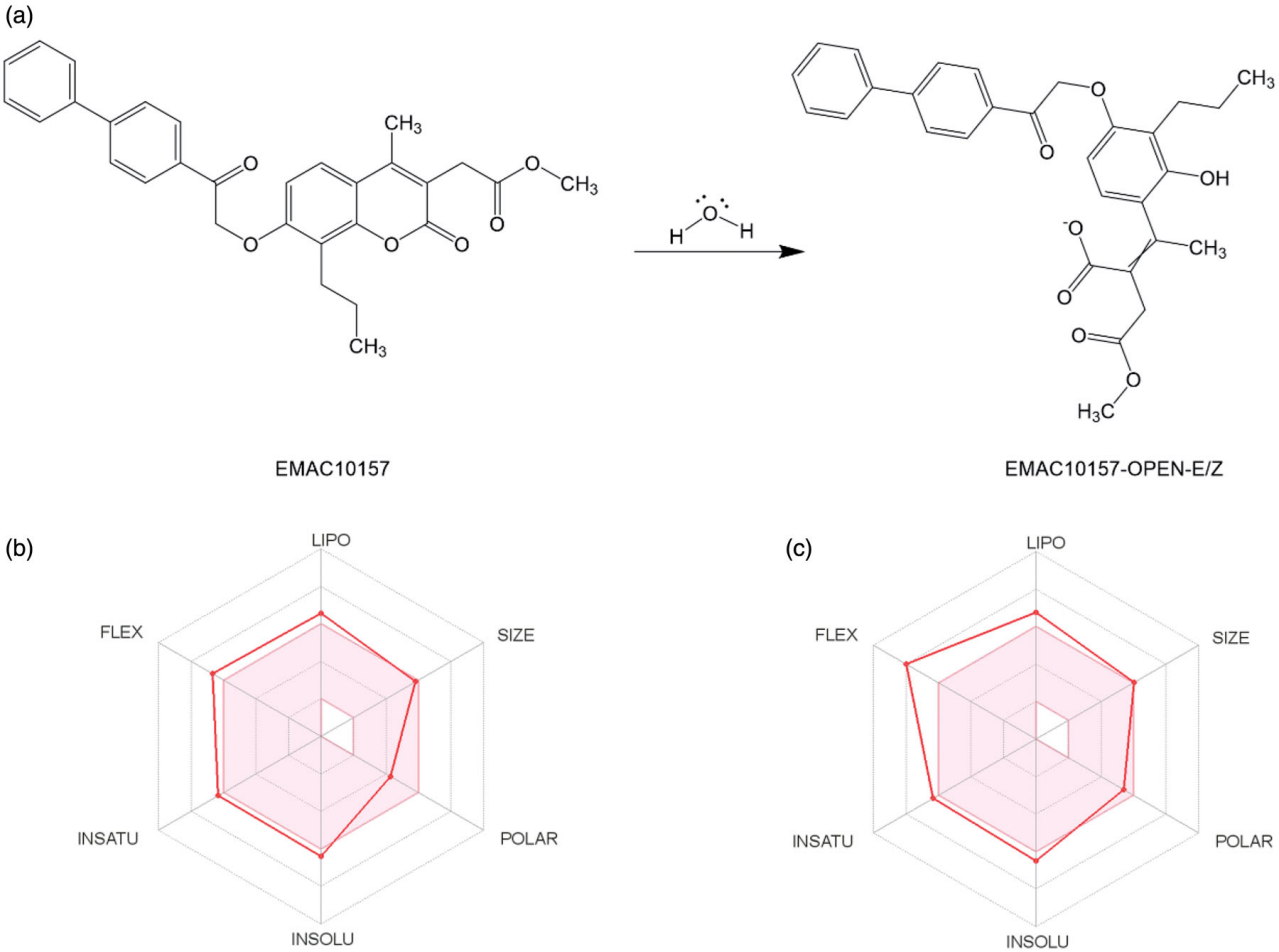
(a) Esterase activity of carbonic anhydrase on compound **EMAC10157g**^34^. (b, c) Oral bioavailability radar profile.

All the synthesised compounds show a propyl substituent in the position 8 or 9 of the coumarin and psoralen nucleus, respectively. As in previously reported derivatives, a methylene carboxylic group was placed in position 3 of the chromene which may lead to the formation of a bidentate chelator of the Zn^2+^ ion in the catalytic pocket, due to the hCA esterase activity^32^^,^^34^^,^^63^ on the dihydropyranone ring, as illustrated in Figure 2.

**Scheme 1.**
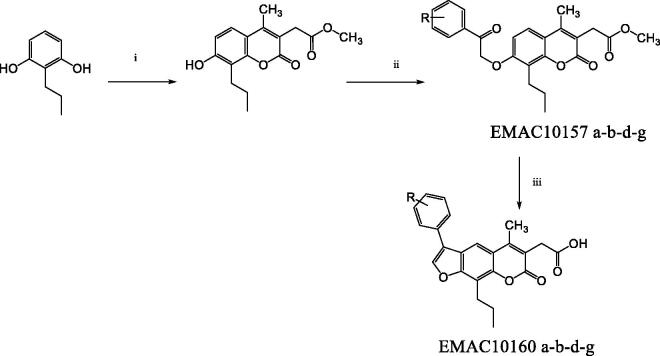
Synthetic pathway to compounds **EMAC10157 a-b-d-g** and **EMAC10160 a-b-d-g**. Reagents and conditions: (i) dimethylacetylsuccinate, H_2_SO_4_ 98% R.T.; (ii) α-halogeno arylketone, dry acetone, K_2_CO_3_, reflux; (iii) NaOH 1 N, reflux.

The synthesis of the new derivatives is reported in Scheme 1. It consists of the H_2_SO_4_ mediated Pechman condensation of dimethylacetylsuccinate and 2-propylresorcinol at room temperature in solvent-free conditions, followed by a Williamson reaction of the phenolic group in the position 7 of the chromene ring with the appropriate α-haloketone in dry acetone and K_2_CO_3_, to generate the asymmetric ether which will lead to the furocoumarin formation by intramolecular electrophilic substitution, mediated by refluxing in NaOH 1 N water solution.

**EMAC10157 a-b-d-g-**and **EMAC10160 a-b-d-g** were characterised employing analytical and spectroscopic methods and the results are summarised in Tables 1 and 2. Compounds were then submitted to enzymatic evaluation towards hCA I, II, IX, and XII. The results are reported in Table 3. Interestingly, none of the new EMAC derivatives exhibited any inhibition activity towards hCA I and II isozymes. On the contrary, all of them are submicromolar inhibitors of the hCA membrane isoforms IX and XII. This behaviour is in agreement with our previous findings and with the generally observed selectivity profile of coumarin derivatives^32^^,^^33^^,^^35^^,^^37^^,^^38^^,^^63^.

**Table 3.** Inhibition data towards hCA I, II, IX, and XII of compounds **EMAC10157a-b-d-g** and **EMAC10160 a-b-d-g**.

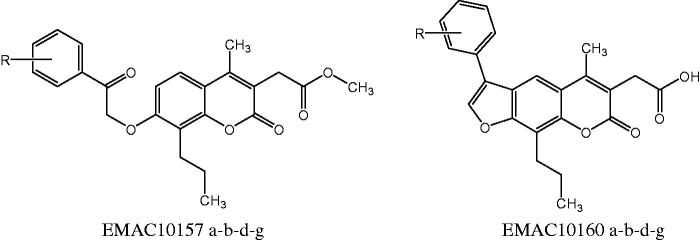					
Compound	R	Ki (nM)*			
		hCA I	hCA II	hCA IX	hCA XII
**EMAC10157a**	4-CH_3_	>10,000	>10,000	247.7	350.3
**EMAC10157b**	4-OCH_3_	>10,000	>10,000	352.7	324.2
**EMAC10157d**	4-F	>10,000	>10,000	239.5	257.1
**EMAC10157g**	4-C_6_H_5_	>10,000	>10,000	135.2	283.1
**EMAC10160a**	4-CH_3_	>10,000	>10,000	467.3	758.1
**EMAC10160b**	4-OCH_3_	>10,000	>10,000	489.3	859.4
**EMAC10160d**	4-F	>10,000	>10,000	379.7	460.0
**EMAC10160g**	4-C_6_H_5_	>10,000	>10,000	397.7	550.0
**AAZ**		250	12	25	5.7

* Standard error (from three different assays) were in the range of plus/minus 10% of the reported values.

Within the tested compounds, **EMAC10157** series was the most active towards the hCA IX and XII isoforms. Therefore, its mechanism of action was investigated in more detail by docking experiments followed by energy minimisation of the obtained complexes.

In particular, considering that an interesting hCA esterase mediated inhibition mechanism was recently reported for coumarin derivatives^32^^,^^33^^,^^63^, we firstly investigated this aspect. Hence, the most promising compound, **EMAC10157g**, was submitted to docking experiments to evaluate if the dihydropyranone ring of the chromene moiety could reach the bottom of the catalytic cavity of hCA II, IX and XII and, therefore, be hydrolysed by the Zn^2+^ activated water molecule, which acts as a very potent nucleophile (Figure 2(a)).

**Figure 3.**
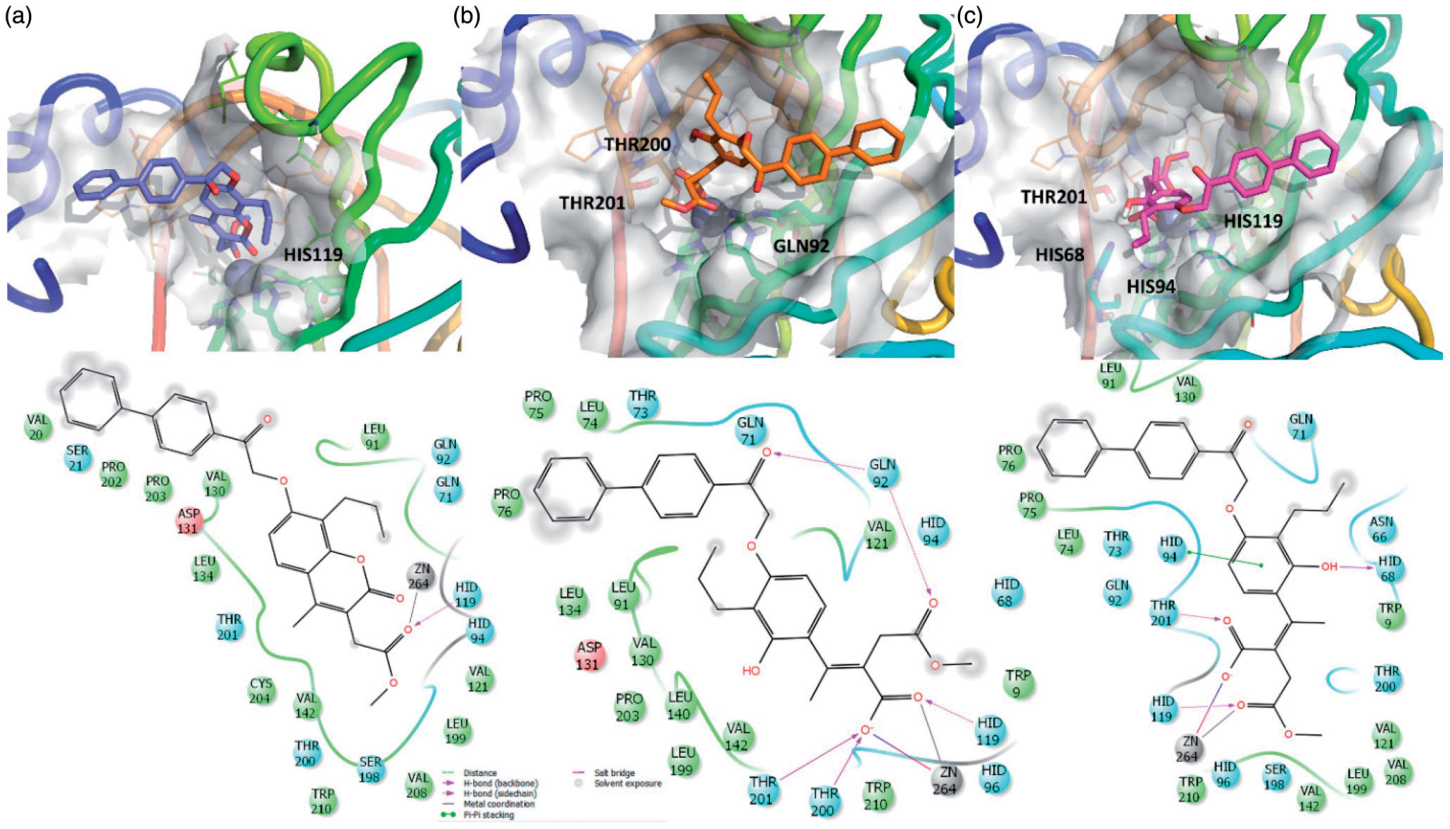
3D representation of the putative binding mode obtained by docking experiments. (a) hCA-IX – **EMAC10157g**, (b) hCA-IX – **EMAC10157g**-openE, and (c) hCA-IX – **EMAC10157g**-openZ and the relative 2D representation of the complexes stabilising interactions with the binding site residues represented with different colour depending on their chemical-physical properties: green, hydrophobic; cyan, polar; violet, positive; red, negative charged residues; grey, metal atoms. Instead, magenta arrows indicate the formation of a hydrogen bond between protein and ligand, while grey lines indicate the interaction with the complexed ion.

Confirming the selectivity profile already observed in previously investigated analogues docking experiment in hCA II enzyme showed that the **EMAC10157g** tail is too bulky to access hCA II cavity, while, in isoforms IX and XII, the dihydropyranone portion of **EMAC10157g** was able to bind close enough to the Zn^2+^ ion (Figures 2(a) and 3(a)) cofactor to undergo the esterase reaction.

**Figure 4.**
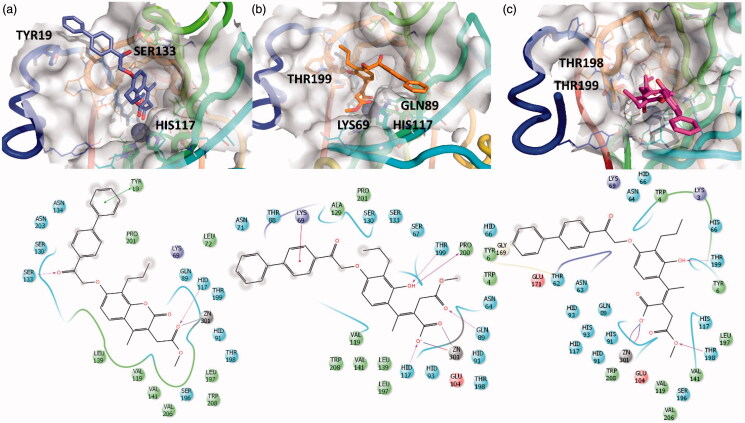
3D representation of the putative binding mode obtained by docking experiments. (a) hCA-XII – **EMAC10157g**, (b) hCA-XII – **EMAC10157g**-openE, and (c) CA-XII- **EMAC10157g**-openZ and the relative 2D representation of the complexes stabilising interactions with the binding site residues.

Hence, the two open cinnamic acid derivative (Figure 2(a)) configurations (E/Z) were subjected to docking experiments to predict the putative binding mode of the hydrolysed forms in hCA IX and hCA XII (Figures 3(b,c) and 4(b,c).

The predicted affinity of the open compounds was estimated to be better than that of the closed ones. What seems important is the Zn^2+^ chelation and the interactions between the newly formed carboxylate moiety and the methyl ester with the surrounding residues in the catalytic site.

The Y shape of the compound, with propyl and -biphenyl-2oxoethoxy moiety being the arms, helps to maintain the selectivity but, ultimately, decreases the activity, compared to previously investigated psoralens^32^.

Biphenyl moiety accommodation is well tolerated due to the wide cavity exposed to the solvent, as shown by other hCA inhibitors with bulky tails. In fact, the interactions between this group with adjacent hydrophobic residues stabilise the complexes.

In summary, the computational methods helped to rationalise the good activity of the investigated compound towards the hCA IX and hCA XII isoform and to suggest a reasonable mechanism of action that could be further investigated to be confirmed. If verified, this compound can be considered as a new prodrug candidate with acceptable oral bioavailability properties (Figure 2(b)), and with good drug-like properties. Nevertheless, considering the encouraging predicted ADME properties^64^ and the activity data, we are further optimising this scaffold.

## Conclusions

We have designed and synthesised a series of *methyl-2-[4-methyl-2-oxo-7–(2-oxo-2-arylethoxyi)-8-propylchromen-3-yl]acetate* and *2–(5-methyl-7-oxo-3-aryl-9-propyl-7H-furo[3,2-g]chromen-6-yl)acetic acid,* and evaluate their activity on hCA I, II, IX, and XII isozymes. As a confirmation of the literature reported chromene derivatives selectivity profile, none of the investigated compounds was able to inhibit the off-target I and II isoforms of hCA. On the contrary, they all inhibit the membrane isozymes hCA IX and XII and further corroborate the reported data on chromene derivatives. Considering the acceptable ADME prediction profile and the high potential of coumarin derivatives, not only as hCA selective inhibitors but also as potential multitarget anticancer agents, these data prompted us to further investigate this scaffold to optimise both the activity and the isozyme selectivity.

## Supplementary Material

Supplemental Material
