# Supplementary material for: Selective inhibition of carbonic anhydrase IX and XII by coumarin and psoralen derivatives
Source: J Enzyme Inhib Med Chem. 2021 Feb 18;36(1):685–92. doi: 10.1080/14756366.2021.1887171 (PMC7899656; doi:10.1080/14756366.2021.1887171)

Supplemental material

Selective inhibition of carbonic anhydrase IX and XII by coumarin and psoralen derivatives

Rita Meleddu[a], Serenella Deplano[a], Elias Maccioni[a]*, Francesco Ortuso[b], Filippo Cottiglia[a], Daniela Secci[a], Alessia Onali[a], Erica Sanna[a], Andrea Angeli[c], Rossella Angius[d], Stefano Alcaro[b], Claudiu T. Supuran[c]*, and Simona Distinto[a].

NMR spectra

Figure S1: EMAC10157a ^{1}H NMR (400 MHz, DMSO)

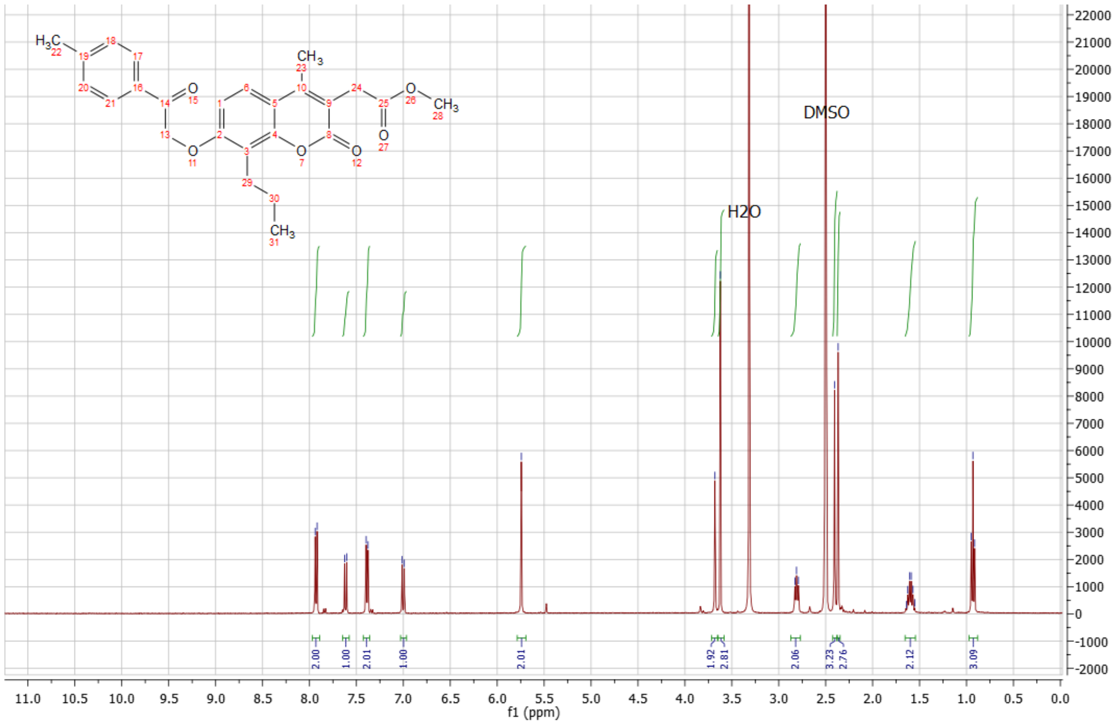

Figure S2: EMAC10157a ^{13}C NMR (100 MHz, DMSO)

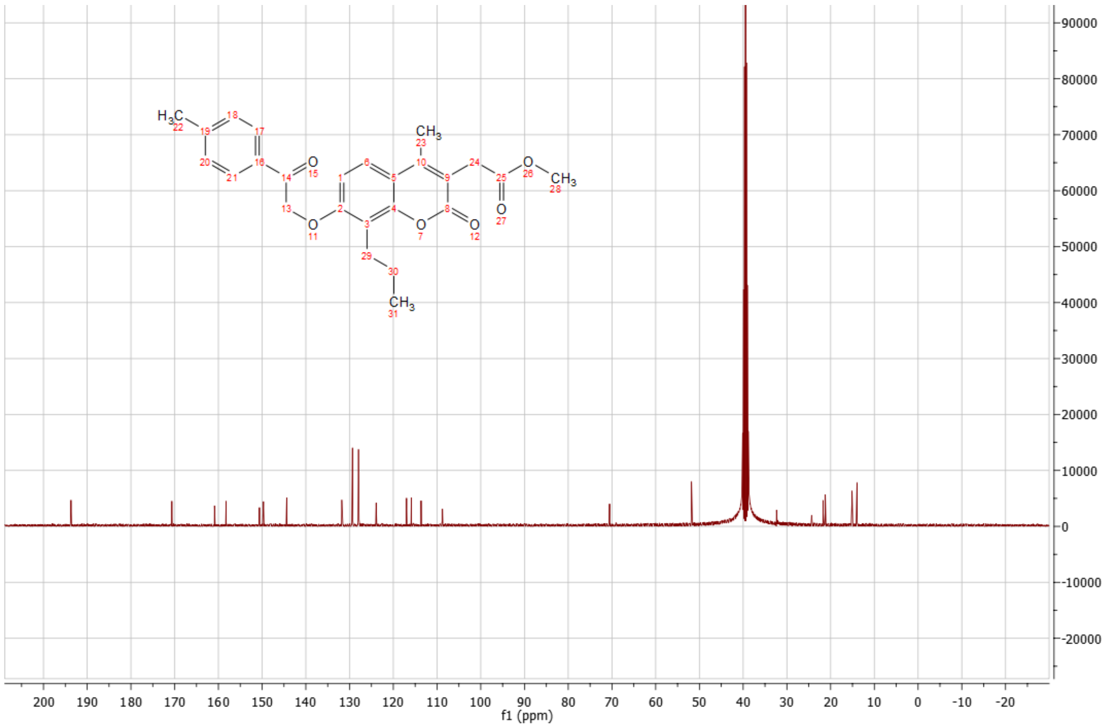

Figure S3: EMAC10157b ^{1}H NMR (400 MHz, DMSO)

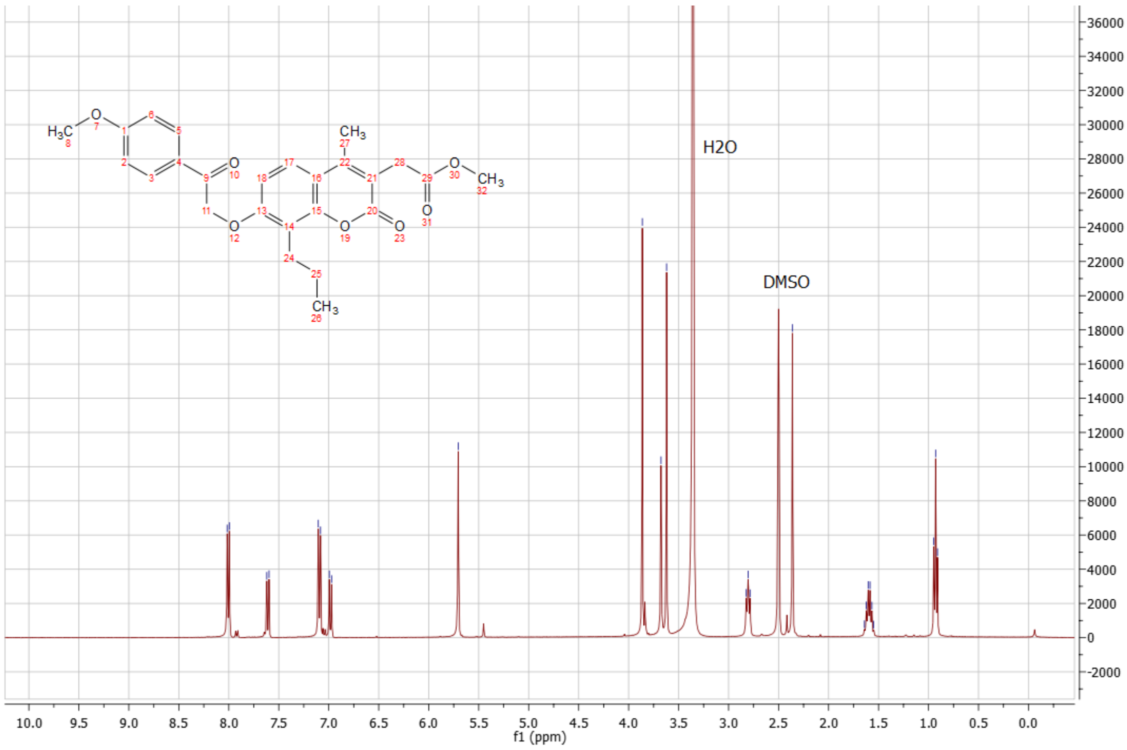

Figure S4: EMAC10157b ^{13}C NMR (100 MHz, DMSO)

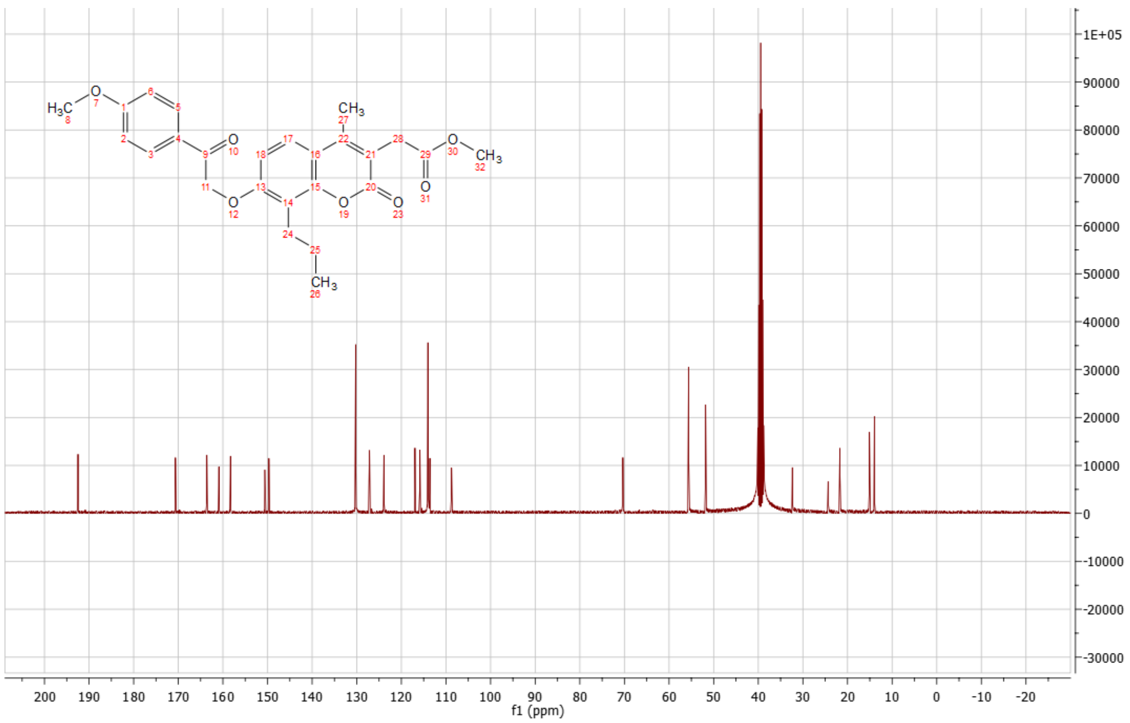

f1 (ppm)

Figure S5: EMAC10157d ^{1}H NMR (400 MHz, DMSO)

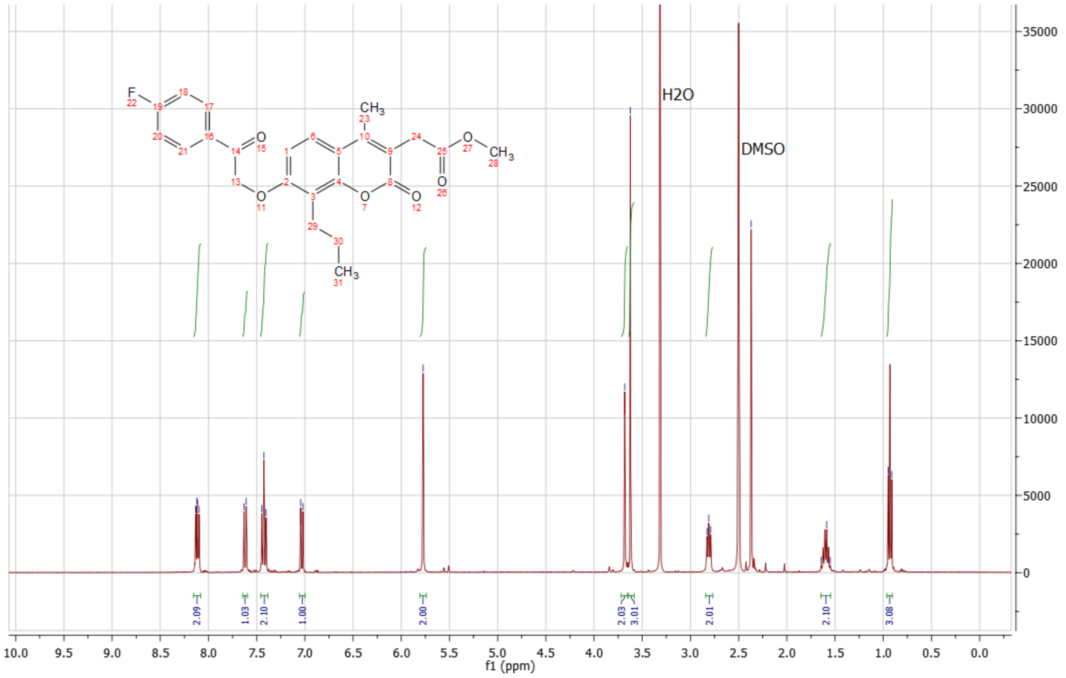

Figure S6: EMAC10157d ^{13}C NMR (100 MHz, DMSO)

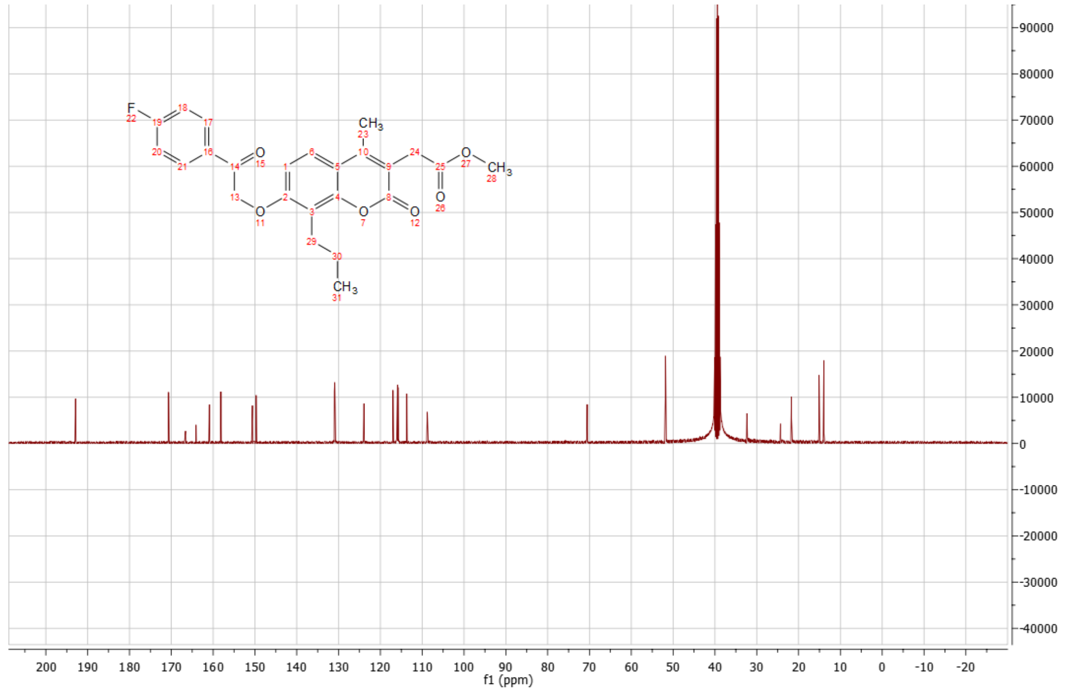

Figure S7: EMAC10157g ^{1}H NMR (400 MHz, DMSO)

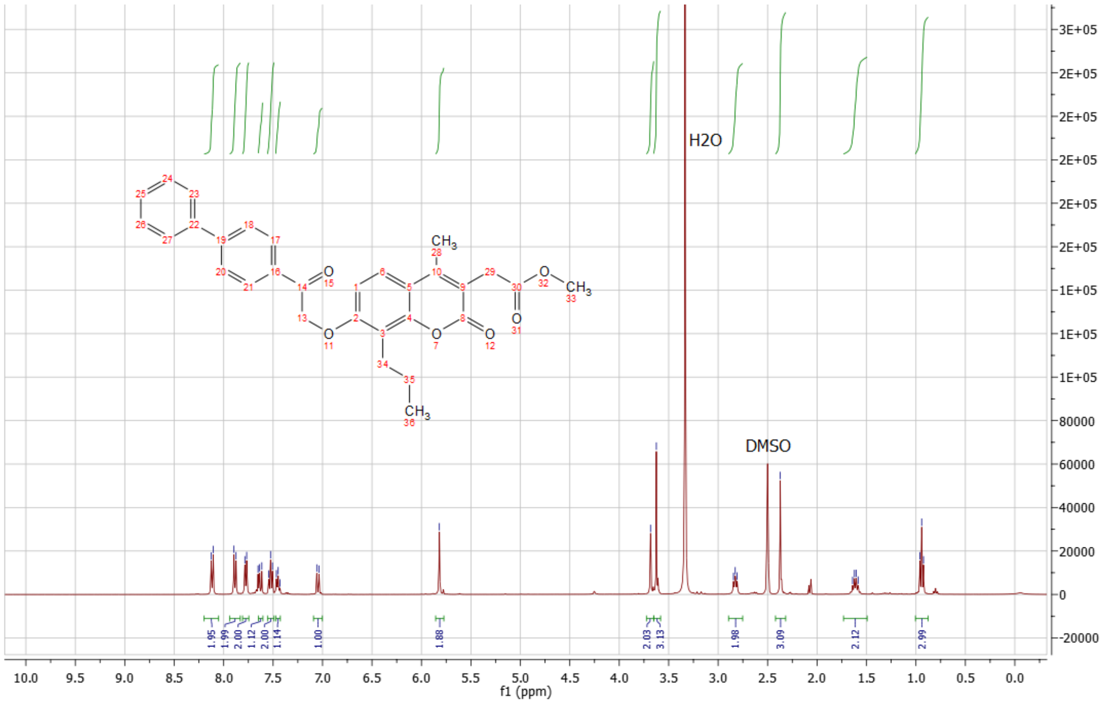

Figure S8: EMAC10157g ^{13}C NMR (100 MHz, DMSO)

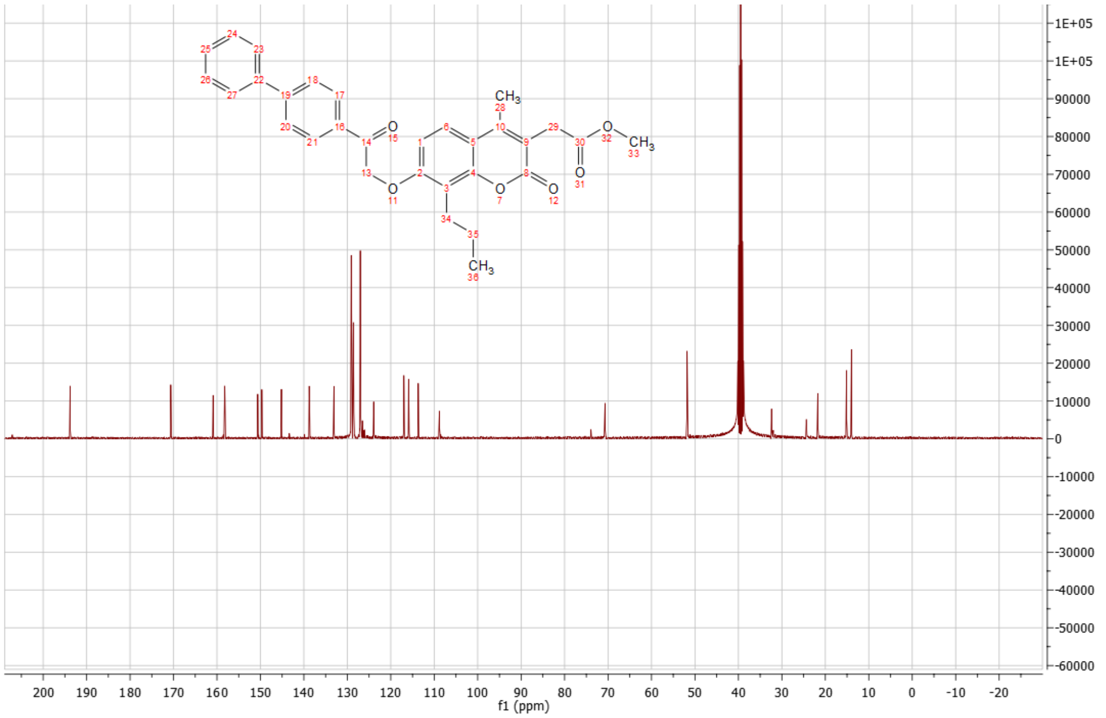

Figure S9: EMAC10160a ^{1}H NMR (400 MHz, DMSO)

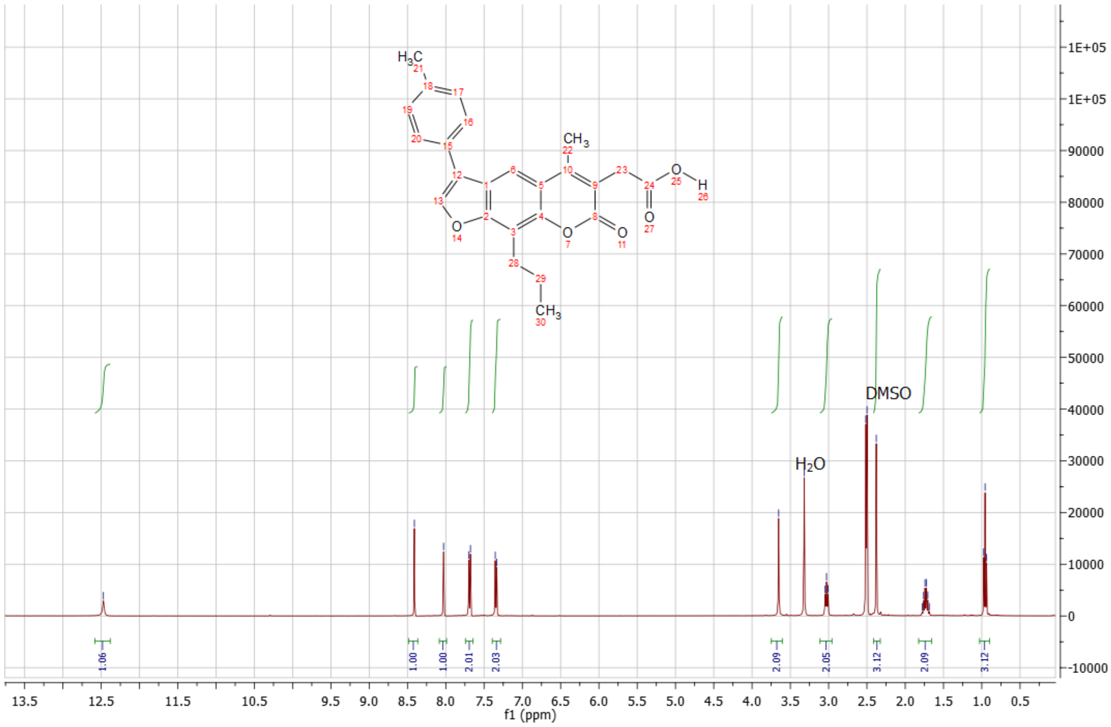

Figure S10: EMAC10160a ^{13}C NMR (100 MHz, DMSO)

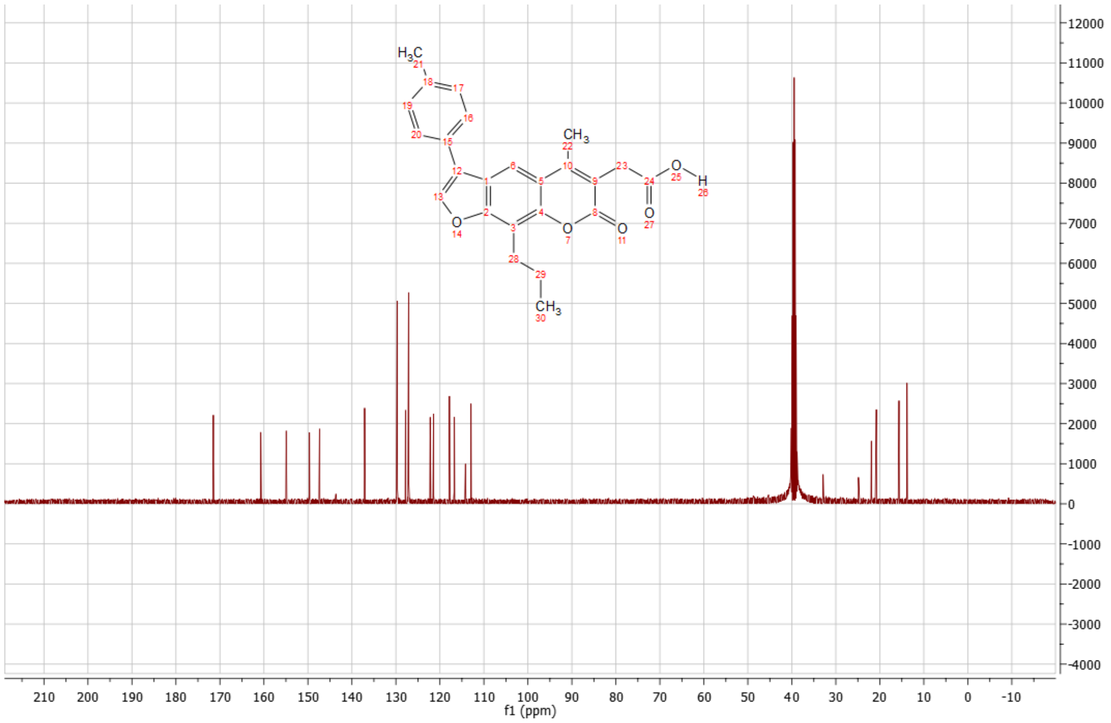

Figure S11: EMAC10160b ^{1}H NMR (400 MHz, DMSO)

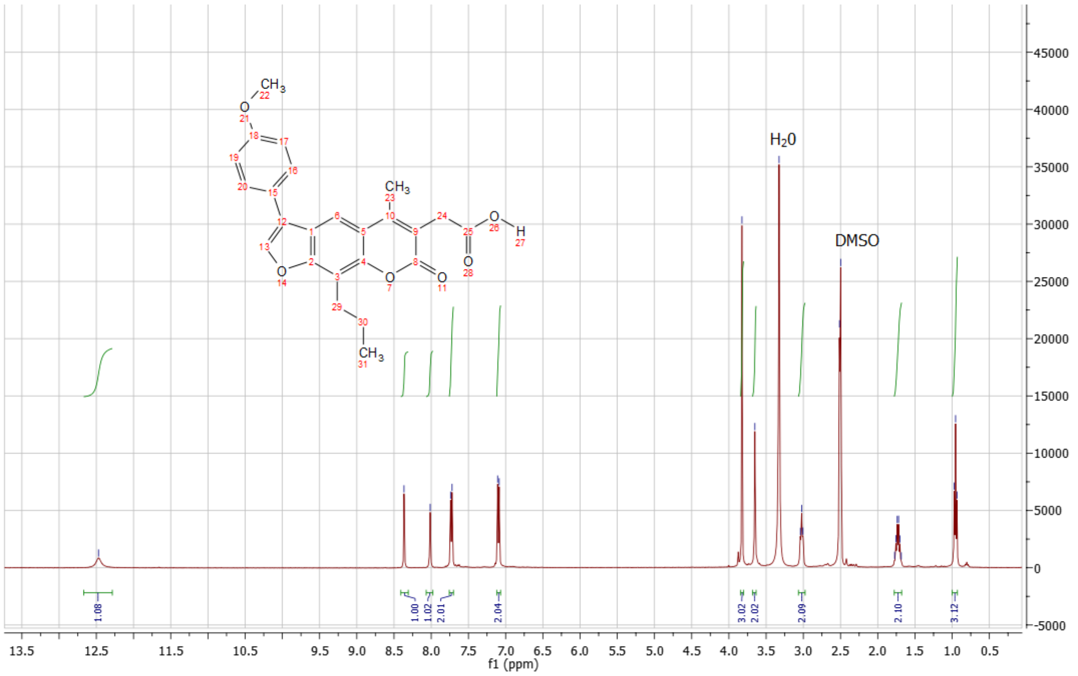

Figure S12: EMAC10160b ^{13}C NMR (100 MHz, DMSO)

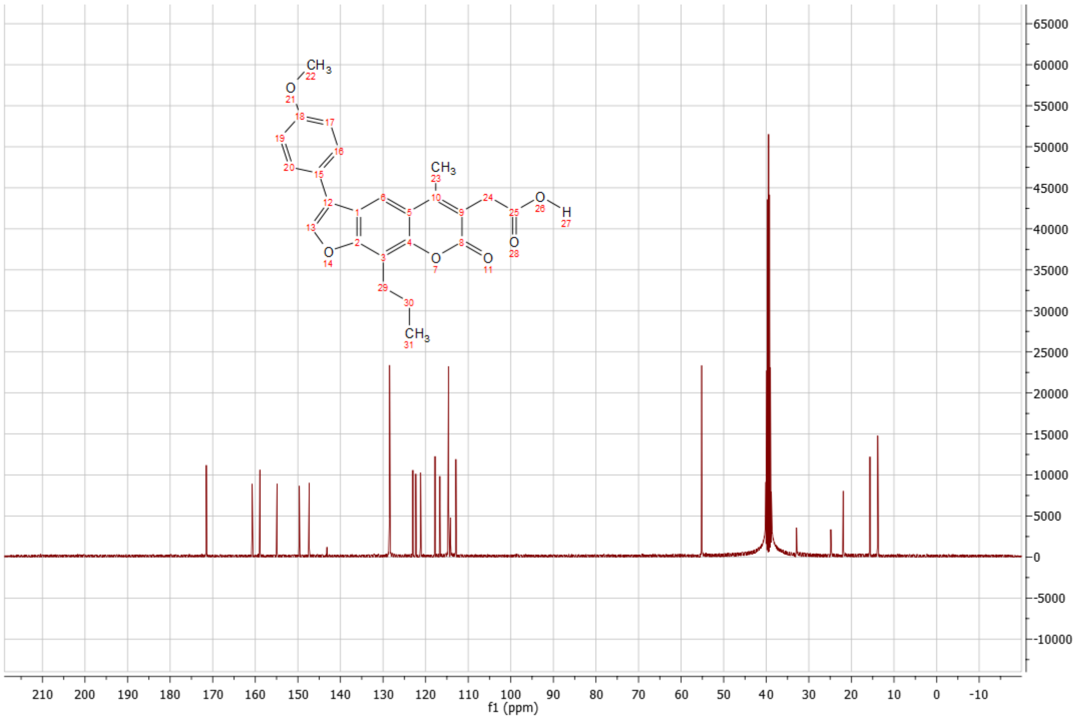

Figure S13: EMAC10160d ^1H NMR (400 MHz, DMSO)

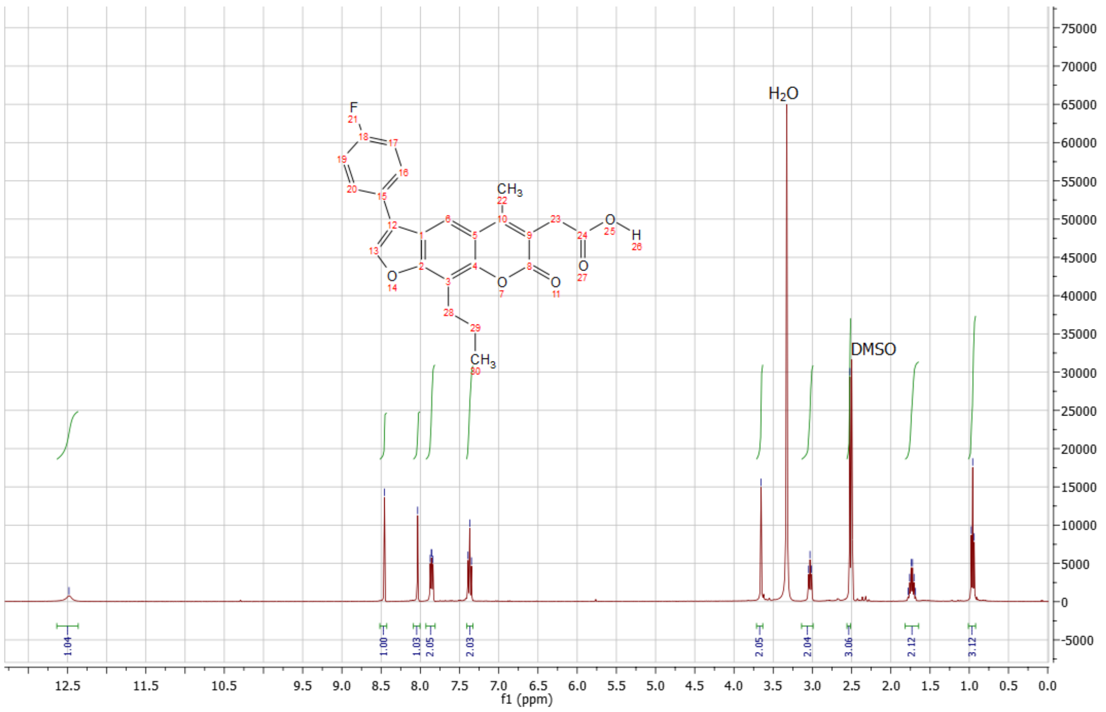

Figure S14: EMAC10160d ^{13}C NMR (100 MHz, DMSO)

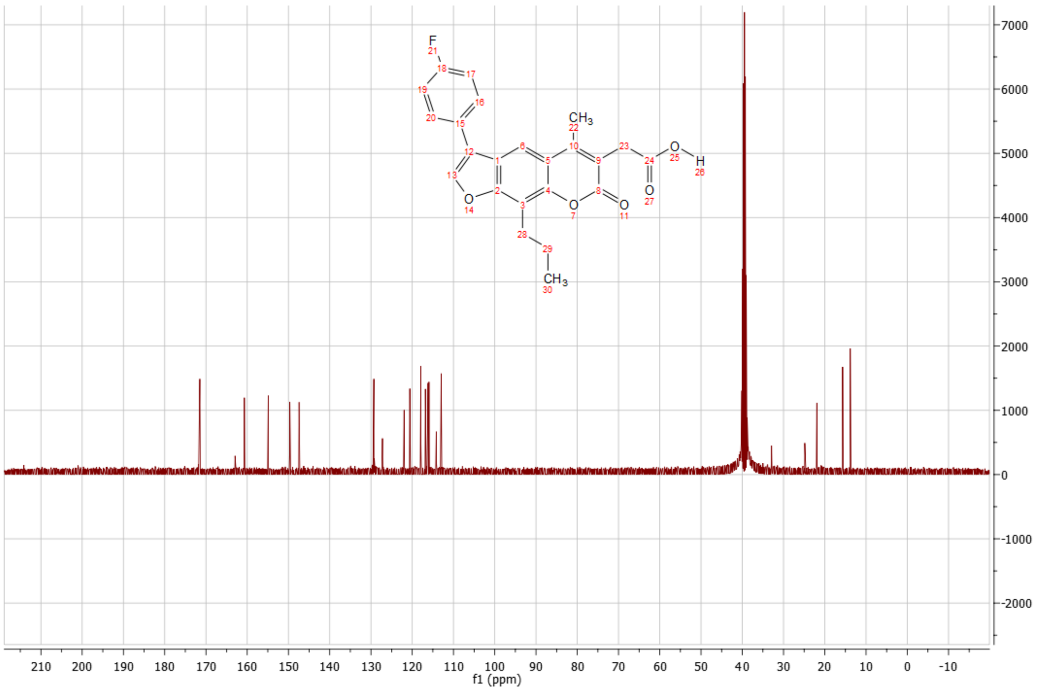

Figure S15: EMAC10160g ^{1}H NMR (400 MHz, DMSO)

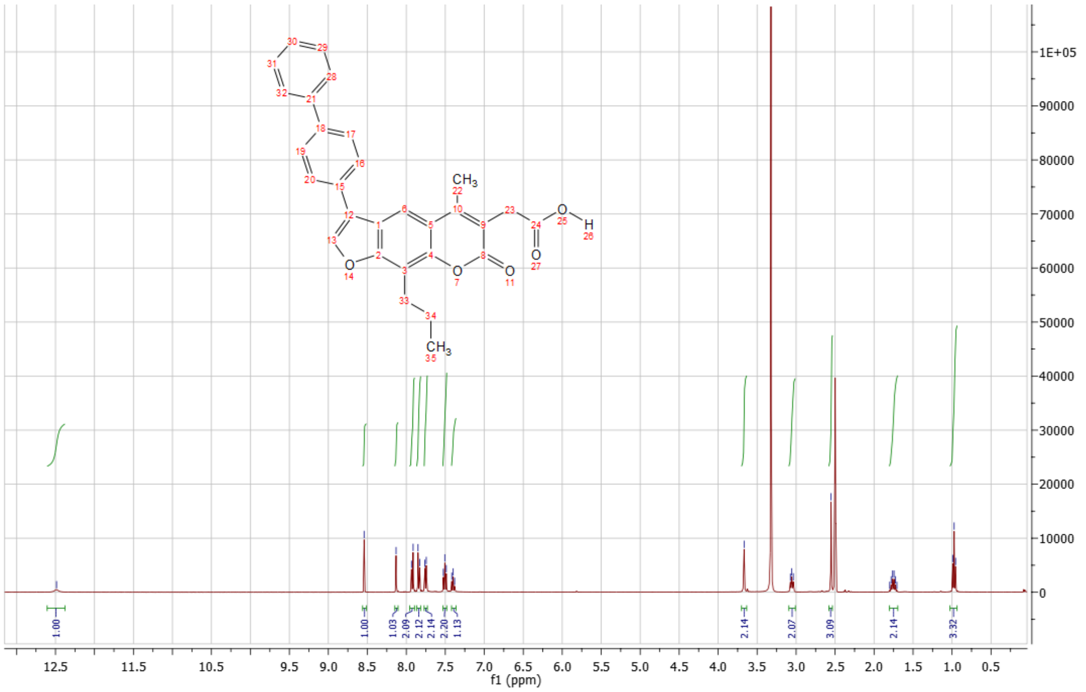

Figure S16: EMAC10160g ^{13}C NMR (100 MHz, DMSO)

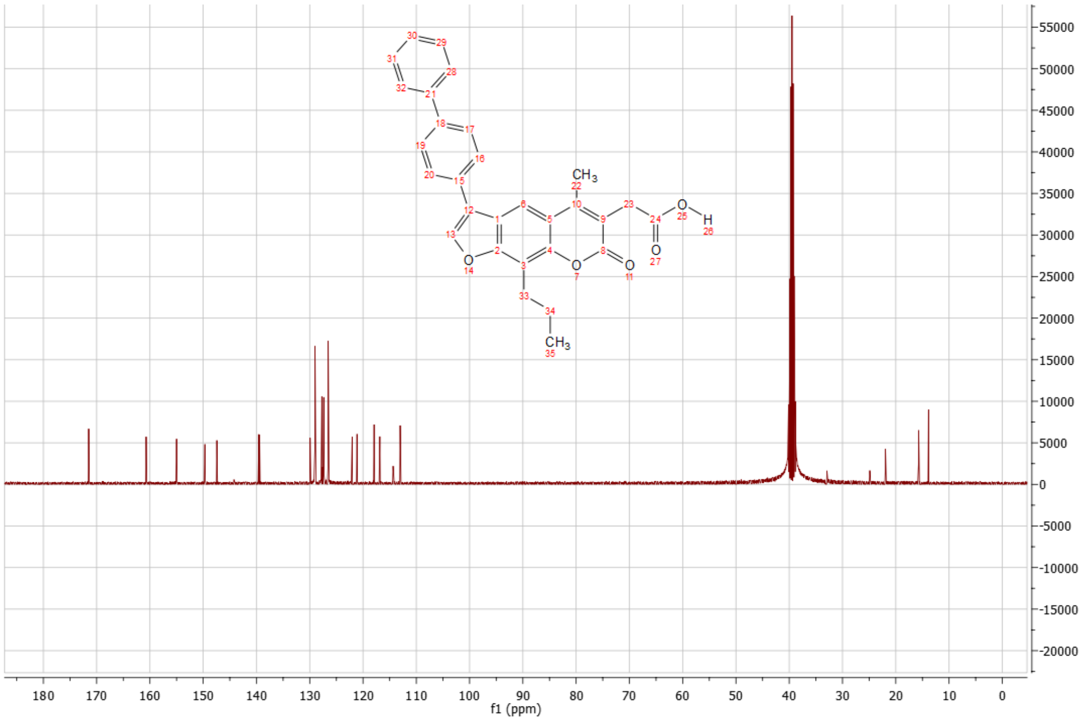

Supplement: Supplemental Material [file IENZ_A_1887171_SM8804.pdf]
